# Intragenomic conflict between transformation and prophage explains taxon-specific DNA acquisition

**DOI:** 10.1093/ismejo/wrag256

**Published:** 2026-10-06

**Authors:** Min Jung Kwun, Nicholas J Croucher

**Affiliations:** MRC Centre for Global Infectious Disease Analysis, Department of Infectious Disease Epidemiology, School of Public Health, Imperial College London, London W12 0BZ, United Kingdom; MRC Centre for Global Infectious Disease Analysis, Department of Infectious Disease Epidemiology, School of Public Health, Imperial College London, London W12 0BZ, United Kingdom; Parasites & Microbes, Wellcome Sanger Institute, Hinxton, Cambridge CB10 1SA, United Kingdom

**Keywords:** transformation, prophage, bacterial evolution, bacterial ecology, recombination, genomics

## Abstract

The activation of many chromosomally integrated mobile genetic elements by RecA-DNA nucleoprotein filaments may represent an adaptation to evading deletion through homologous recombination. Consistent with bacterial transformation facilitating such eliminations of inserted viral DNA, cross-taxa comparisons found isolates’ prophage content was negatively associated with homologous recombination rates. Assaying the effect of the composition of the extracellular DNA pool on transformation of a lysogen demonstrated that DNA from conspecific donors drove both prophage activation and deletion, whereas DNA originating from different species triggered only prophage activation. Accordingly, rates of horizontal DNA transfer mechanisms varied across bacterial ecologies, with homologous recombination common relative to prophage accumulation when DNA originated from fewer donor species.

## Graphical abstract

**Graphical abstract:**
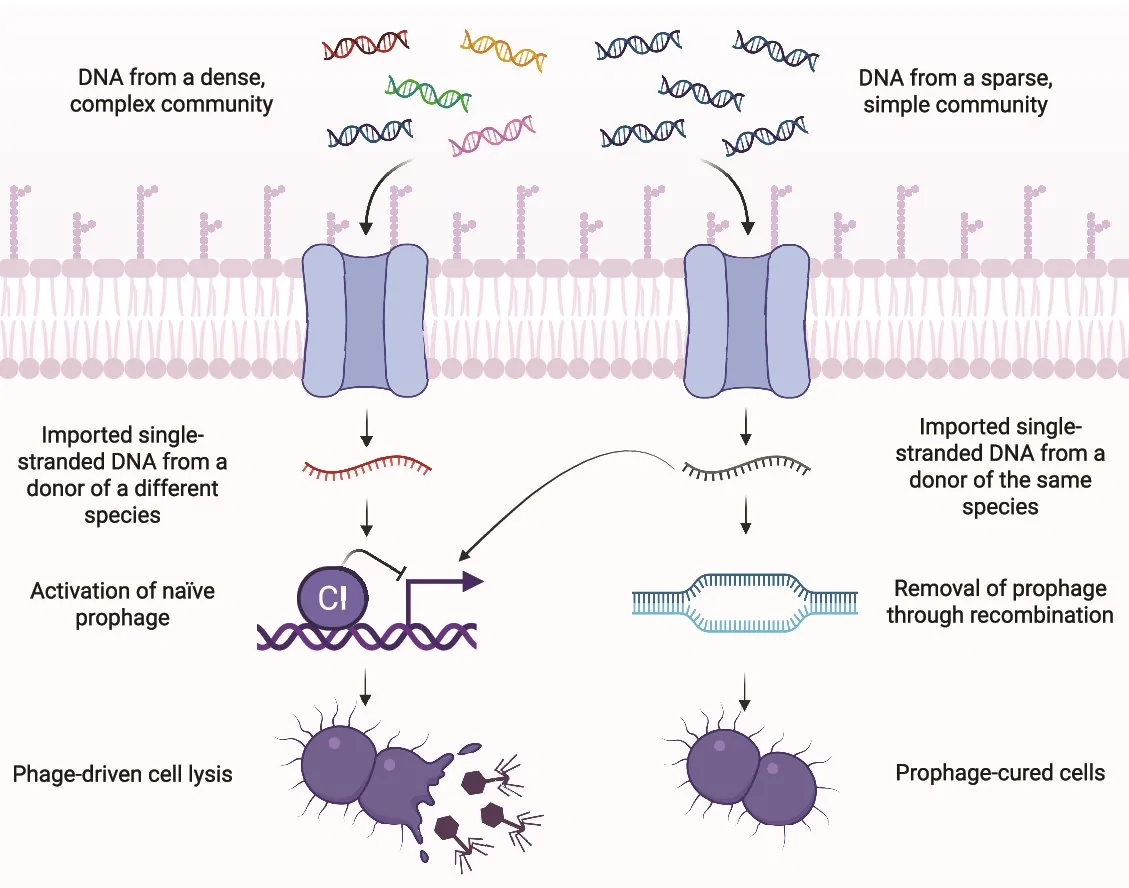

Transformation occurs when prokaryotes import exogenous DNA via the competence machinery and integrate it into their genomes through homologous recombination (HR). Despite the conservation of the competence machinery, it is difficult to activate in many bacteria, and even naturally transformable species limit the DNA they acquire [1]. Some preferentially import sequences containing species-specific uptake motifs, whereas others couple transient activation of competence with fratricidal mechanisms that release DNA from conspecific donors [1]. Both mechanisms ensure that DNA exchange through transformation is primarily intraspecific [2].

Although the primary function of the competence machinery remains controversial [3–5], such behaviour is consistent with transformation driving chromosomal curing, the elimination of chromosomally integrated mobile genetic elements (MGEs) by HR [3]. Transformation is efficient at deleting pneumococcal prophages, despite “naive” prophages activating and lysing the host bacterial cell when they detect RecA-DNA nucleoprotein filaments generated by the competence machinery [6]. This response is typically driven by protein–protein interactions with prophage repressors, as observed across a broad taxonomic distribution [7]. By contrast, the efficiency of HR is driven by DNA–DNA interactions, and declines exponentially with divergence between donor and recipient [8]. Hence the preference for intraspecific donor DNA may indicate selection against molecules that would activate naïve prophage, but not be able to delete such elements.

This proposed differential sensitivity to donor DNA sequence can be tested using the *Streptococcus pneumoniae* RMV8 prophage–host model system [6]. This genotype is naturally transformable with DNA from any source, either at a low level spontaneously, or at a higher level following competence induction by administration of exogenous competence stimulating peptide (CSP), which upregulates the machinery required for DNA import and recombination [9]. It also harbours a single naïve prophage that can be induced by the importation of pneumococcal DNA by the competence machinery, ϕRMV8 [6], and a phage-related chromosomal island, PRCI*_malA_* [10]. To assay the extracellular release of both ϕRMV8 and PRCI*_malA_*, quantitative polymerase chain reactions (PCRs) were used to measure the abundance of their *attP* sites, specific to the circularized forms of these elements, in culture supernatants (see Supplementary Materials & Methods; Table S1). Control experiments showed CSP increased extracellular ϕRMV8 *attP* site concentration ~ 20-fold relative to chromosomal DNA, but had little effect on PRCI*_malA_ attP* levels (Fig. S1). However, both the ϕRMV8 and PRCI*_malA_ attP* sites became more DNase I resistant 2 h post-CSP administration, indicating both genomes were being packaged within virions following competence induction [11].

To test how competence-associated prophage induction was affected by the import of DNA from divergent sources, replicating the situation in multi-species communities, CSP was administered in conjunction with DNA mixtures containing 1%–99% *S. pneumoniae* RMV8-derived DNA (in which ϕRMV8 was replaced with a kanamycin-resistance marker, and PRCI*_malA_* was replaced with a chloramphenicol-resistance marker), with the remainder comprising *Escherichia coli* genomic DNA. Independent of the DNA composition, CSP induced a ~ 20-fold increase in ϕRMV8 release after two hours (Fig. 1A, Fig. S2). This activation depended upon the exogenous DNA, as the addition of DNase I abrogated the effect, and the DNA released by pneumococcal growth (Figs S3-S4) triggered a slower release of ϕRMV8, in the period 2–4 hours post-CSP. Hence induction of ϕRMV8 in competent pneumococci was DNA-dependent but sequence-independent.

**Figure 1:**
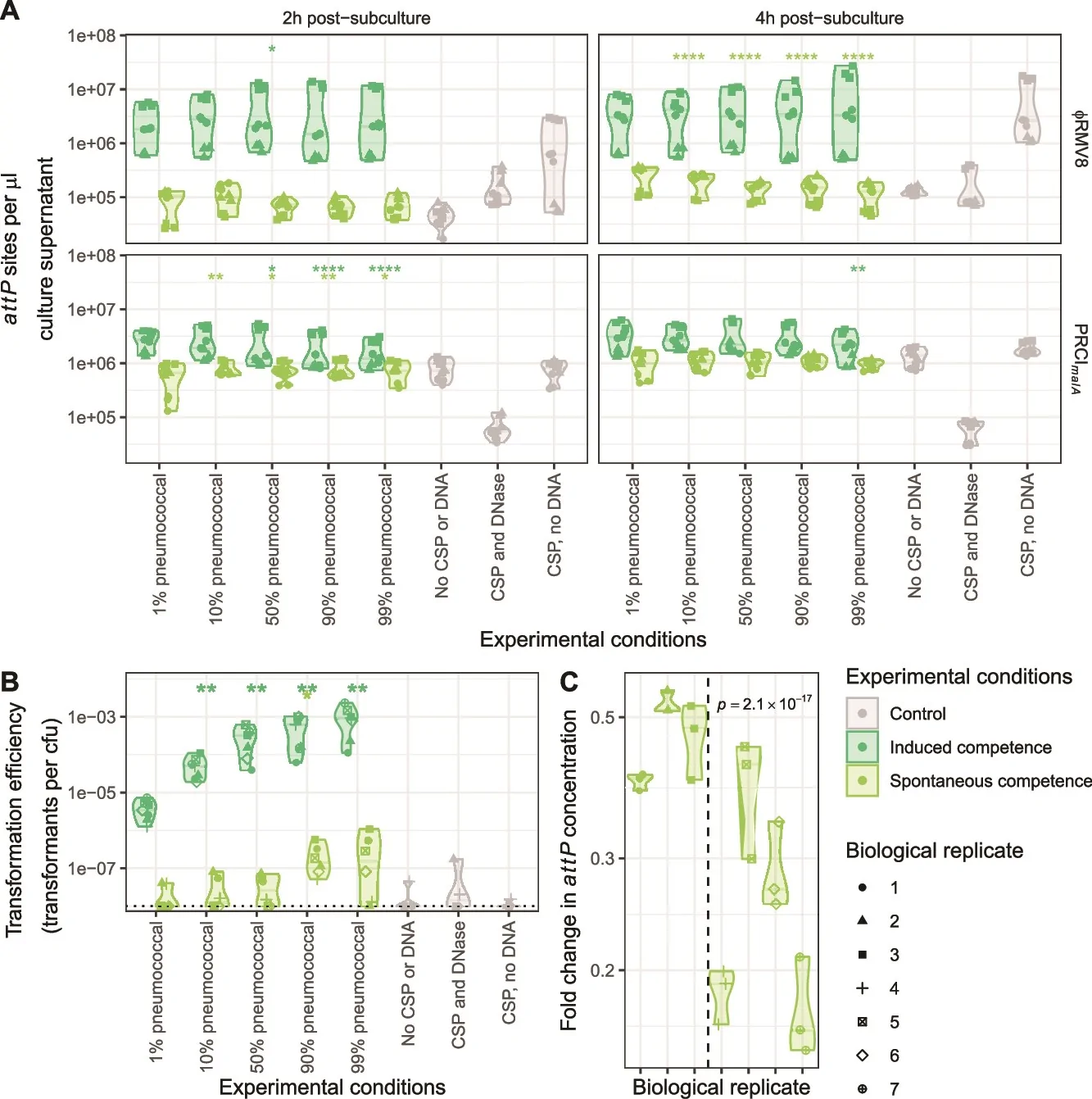
Experiments testing the ability of exogenous DNA to activate and delete MGEs. (A) Violin plots showing the concentration of *attP* sites in culture supernatant, used to quantify the release of MGEs from their host cells, for ɸRMV8 and PRCI*_malA_* either two hours or four hours after subculturing into the specified experimental conditions. Each violin has a horizontal line at the median value. Each point represents one of three technical replicate measurements of three independent biological replicates, as indicated by its shape. The significances of differences between the results from transformations using 1% pneumococcal DNA, and experiments using higher pneumococcal DNA proportions, were tested using pairwise generalized linear mixed effects model fits that accounted for the structuring of the data into biological and technical replicates (see supplementary methods). The *P* values (separately adjusted using a Holm-Bonferroni correction for each combination of MGE, timepoint and transformation type) are indicated above the corresponding data points by asterisks: ^*^ indicates *P* < 0.05; ^**^ indicates *P* < 0.01; ^***^ indicates *P* < 0.001, and ^****^ indicates *P* < 0.0001. (B) Violin plots showing the rates at which ɸRMV8 was replaced by a kanamycin-resistance marker. Each point represents an independent biological replicate. The significances of differences between the results from transformations using 1% pneumococcal DNA, and experiments using higher pneumococcal DNA proportions, were tested using two-sided Wilcoxon rank-sum tests. The *P* values are indicated by the asterisks at the top of the panel, as in panel (A), following a Holm-Bonferroni correction. (C) Violin plots showing the fold change in the concentration of ɸRMV8 *attP* in the culture supernatant when spontaneously competent cells were transformed with 99% pneumococcal DNA relative to 1% pneumococcal DNA. The first three biological replicates are represented in panel (A); additional biological replicates, shown to the right of the dashed line, represent subsequent independent experiments. The annotated *P* value represents the level of significance at which the null hypothesis of no change in *attP* levels across different pneumococcal DNA proportions can be rejected, using the same approach as in panel (A).

The rate at which prophages were replaced by the kanamycin-resistance marker during CSP-induced transformation was also assayed (Fig. 1B). This was highly sensitive to the composition of the exogenous DNA mix, rising ~210-fold as the proportion of pneumococcal DNA increased from 1% to 90%. The maximal efficiency was ~10^−3^ transformants per cell, suggesting ~10% of prophage were deleted from the ~1% of pneumococci induced to become competent [12]. Hence the competence machinery’s propensity to specifically acquire conspecific donor DNA maximizes chromosomal curing relative to prophage activation.

Consistent with the increased deletion of MGEs by transformation with conspecific DNA, the *attP* sites were significantly reduced in concentration as the pneumococcal contribution to the extracellular DNA mixture increased (Fig. 1B). When competence was spontaneous, the ϕRMV8 *attP* sites declined 50%–75% as the pneumococcal DNA rose from 1% to 99% of the extracellular DNA mixture. Four subsequent independent replicates validated this observation (Fig. 1C). This likely reflected prophage deletion being most effective under the optimal conditions of a small subpopulation of pneumococci becoming competent in the presence of a saturating concentration of DNA from uninfected conspecific donors [3]. Such an estimated efficacy of curing suggested it was likely to be advantageous to monolysogenic bacteria, but unlikely to remove all prophage from a polylysogen without activating at least one virus. As conflict between HR and prophage is hypothesized to be universal within prokaryotes, this implied the maximum rate of transformation would be lower in bacterial taxa that are commonly polylysogenic.

This was tested across 23 taxa either demonstrated to be naturally transformable in the laboratory [13], or known to cause substantial burdens of antibiotic-resistant disease [14] (Table S2). Multi-locus sequence typing data [15] were used to quantify the probability of diversification via HR by analysing the number of base substitutions in loci differentiating single locus variants using a generalised linear mixed effects model (Figs S5 and S6). In parallel, complete genome sequences were used to identify prophage likely to cause cell lysis in response to RecA-nucleoprotein filaments (Figs S7-S11). Joint analysis of both quantities using a generalized linear model identified a significant negative relationship between the frequency of HR and prophage content (Figs 2A and S12-S14). HR was highest in naturally transformable taxa, which were less likely to be polylysogenic (Figs S15-S16). This suggests that once bacteria are infected by multiple prophages, importing DNA for HR becomes riskier, as the viruses will compete to be the first to activate and lyse the host cell. *Acinetobacter baumannii, Bacillus subtilis*, and *Haemophilus influenzae* were outliers in being both naturally transformable and commonly polylysogenic. Correspondingly, prophage infection has been negatively associated with transformation in these bacteria [16–18], suggesting the inverse relationship is also evident within species.

**Figure 2:**
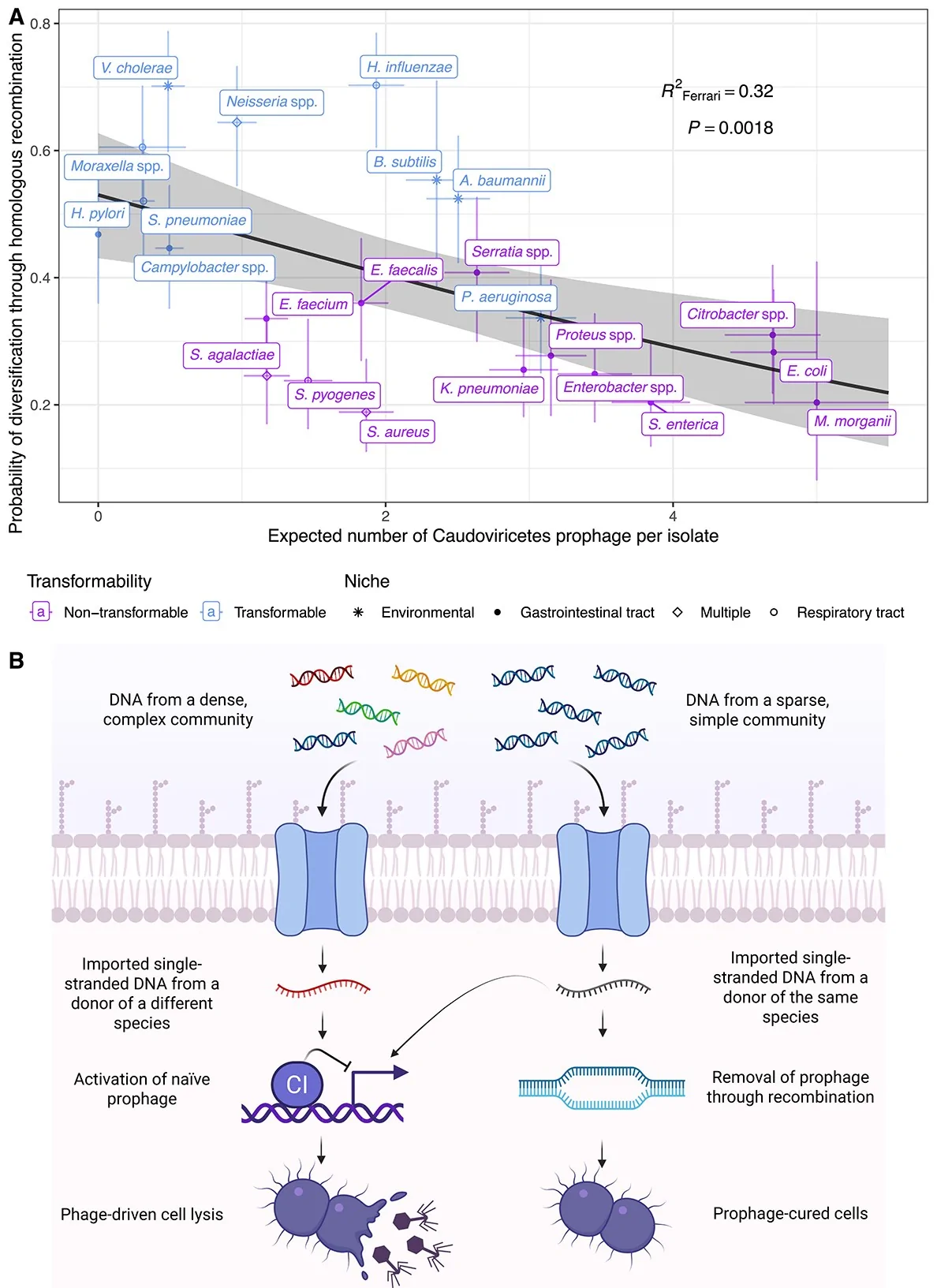
A framework for explaining the relationship between bacterial ecology, transformation, and prophage across microbiomes. (A) Scatterplot showing the relationship between the expected numbers of prophage in a taxon representative’s genome, inferred from complete genomes, and the probability of it diversifying through HR rather than point mutation, inferred from multi-locus sequence typing data (Table S2). The points represent maximum likelihood estimates, and the error bars show 95% confidence intervals. The solid line shows the values predicted by a generalized linear model of the relationship between the displayed properties, with the grey shaded area showing the 95% confidence intervals. The annotated *R*^2^ and *P* values show the strength and significance of the inferred relationship between the properties compared in the graph. Points are coloured according to whether each taxon is known to be naturally transformable *in vitro*, with the shape of the point describing the ecology of the taxon (Table S5). (B) Summary of the proposed hypothesis linking bacterial ecology with the intragenomic conflict between transformation and MGEs.

The separation of taxa along the spectrum from high HR to high prophage content suggested an ecological influence on bacterial evolution. Transformation was less common in the intestine (Figs S15-S16), which is dense with a diverse mixture of species, and more common in the nasopharynx, where the microbiome is less complex [19]. This suggests chromosomal curing is most effective in simpler communities, where it is easier to primarily import DNA from conspecific donors (Fig. 2B). Hence in complex communities, bacteria may increase the specificity of DNA import, or downregulate transformation. A reduction in HR would result in an accumulation of more stable MGEs that are likely under stronger selection to co-evolve with their longer-term hosts [20]. Therefore, the diversity of prokaryotic ecology is likely to modulate the relationships between host cells and temperate phage, with important consequences on bacterial population structure and evolution.

## Supplementary Material

Recombination_conflict_supplementary_datasets_ISME_upload

## Data Availability

All code and data can be accessed from https://github.com/nickjcroucher/recombination-conflict.

## References

[1] Johnston C, Martin B, Fichant G., et al. Bacterial transformation: distribution, shared mechanisms and divergent control. *Nat Rev Microbiol* 2014;12:181–96. 10.1038/nrmicro3199 24509783

[2] Suckow G, Seitz P, Blokesch M. Quorum sensing contributes to natural transformation of *Vibrio cholerae* in a species-specific manner. *J Bacteriol* 2011;193:4914–24. 10.1128/JB.05396-11 PMC3165701 21784943

[3] Croucher NJ, Mostowy R, Wymant C., et al. Horizontal DNA transfer mechanisms of bacteria as weapons of intragenomic conflict. *PLoS Biol* 2016;14:e1002394. 10.1371/journal.pbio.1002394 PMC4774983 26934590

[4] Arnold BJ, Huang IT, Hanage WP. Horizontal gene transfer and adaptive evolution in bacteria. *Nat Rev Microbiol* 2022;20:206–18. 10.1038/s41579-021-00650-4 34773098

[5] Redfield RJ . Genes for breakfast: the have-your-cake and-eat-it-too of bacterial transformation. *J Hered* 1993;84:400–4. 10.1093/oxfordjournals.jhered.a111361 8409360

[6] Kwun MJ, Ion AV, Apagyi KJ., et al. Chromosomal curing drives an arms race between bacterial transformation and prophage. *Mol Biol Evol* 2025;42:msaf259. 10.1093/molbev/msaf259 PMC12637031 41269752

[7] Dahlman S, Avellaneda-Franco L, Rutten EL., et al. Isolation, engineering and ecology of temperate phages from the human gut. *Nature.* 2025;647:698–705. 10.1038/s41586-025-09614-7 PMC12629997 41094135

[8] Roberts MS, Cohan FM. The effect of DNA sequence divergence on sexual isolation in bacillus. *Genetics.* 1993;134:401–8. 10.1093/genetics/134.2.401 PMC1205484 8325477

[9] Kwun MJ, Ion AV, Cheng H-C., et al. Post-vaccine epidemiology of serotype 3 pneumococci identifies transformation inhibition through prophage-driven alteration of a non-coding RNA. *Genome Med* 2022;14:144. 10.1186/s13073-022-01147-2 PMC9764711 36539881

[10] Lim TY, Horsfield ST, Troman CM., et al. Pore-C sequencing identifies episome-driven chromosome conformation perturbations differentiating pneumococcal epigenetic variants. *PLoS Pathog* 2025;21:e1013392. 10.1371/journal.ppat.1013392 PMC12416852 40811750

[11] Zhang Z, Kottadiel VI, Vafabakhsh R., et al. A promiscuous DNA packaging machine from bacteriophage T4. *PLoS Biol* 2011;9:e1000592. 10.1371/journal.pbio.1000592 PMC3039672 21358801

[12] Kwun MJ, Ion AV, Oggioni MR., et al. Diverse regulatory pathways modulate bet hedging of competence induction in epigenetically-differentiated phase variants of *Streptococcus pneumoniae*. *Nucleic Acids Res* 2023;51:10375–94. 10.1093/nar/gkad760 PMC10602874 37757859

[13] Johnsborg O, Eldholm V, Håvarstein LS. Natural genetic transformation: prevalence, mechanisms and function. *Res Microbiol* 2007;158:767–78. 10.1016/j.resmic.2007.09.004 17997281

[14] Murray CJL, Ikuta KS, Sharara F., et al. Global burden of bacterial antimicrobial resistance in 2019: a systematic analysis. *Lancet* 2022;399:629–55. 10.1016/S0140-6736(21)02724-0 PMC8841637 35065702

[15] Maiden MCJ, Bygraves JA, Feil E., et al. Multilocus sequence typing: a portable approach to the identification of clones within populations of pathogenic microorganisms. *Proc Natl Acad Sci USA* 1998;95:3140–5. 10.1073/pnas.95.6.3140 PMC19708 9501229

[16] Setlow Jane K, Boling ME, Allison DP., et al. Relationship between prophage induction and transformation in *Haemophilus influenzae*. *J Bacteriol* 1973;115:153–61. 10.1128/jb.115.1.153-161.1973 PMC246225 4541535

[17] Yasbin RE, Young FE. The influence of temperate bacteriophage φ105 on transformation and transfection in *Bacillus subtilis*. *Biochem Biophys Res Commun* 1972;47:365–71. 10.1016/0006-291X(72)90722-X 4196974

[18] Mazzamurro F, Chirakadavil JB, Durieux I., et al. Intragenomic conflicts with plasmids and chromosomal mobile genetic elements drive the evolution of natural transformation within species. *PLoS Biol* 2024;22:e3002814. 10.1371/journal.pbio.3002814 PMC11472951 39401218

[19] Huttenhower C, Gevers D, Knight R., et al. Structure, function and diversity of the healthy human microbiome. *Nature.* 2012;486:207–14. 10.1038/nature11234 PMC3564958 22699609

[20] Canchaya C, Fournous G, Brüssow H. The impact of prophages on bacterial chromosomes. *Mol Microbiol* 2004;53:9–18. 10.1111/j.1365-2958.2004.04113.x 15225299

